# Evaluation of a community of practice for speech-language pathologists in aphasia rehabilitation: a logic analysis

**DOI:** 10.1186/s12913-019-4338-0

**Published:** 2019-07-29

**Authors:** Christine Alary Gauvreau, Guylaine Le Dorze, Dahlia Kairy, Claire Croteau

**Affiliations:** 1 School of Speech-Language Pathology and Audiology, Faculty of Medicine, Université de Montréal, Montreal, Quebec Canada; 2 School of Rehabilitation, Faculty of Medicine, Université de Montréal, Montreal, Quebec Canada; 3 Centre for Interdisciplinary Research in Rehabilitation of Greater Montreal (CRIR), Montreal, Quebec Canada; 4 Jewish Rehabilitation Hospital, Centre intégré de santé et de services sociaux de Laval, Laval, Quebec Canada

**Keywords:** Knowledge transfer, Community of practice, Continuing education, Speech-language pathology, Aphasia, Rehabilitation, Participation, Logic analysis, Qualitative thematic analysis

## Abstract

**Background:**

Aphasia is a communication disorder affecting participation. Although there are evidence-based practice recommendations about participation and aphasia rehabilitation, it may be challenging for speech-language pathologists to ensure that rehabilitation activities have an impact on the person’s participation, in part due to time limitations. Participation remains limited after rehabilitation for persons who have aphasia. Communities of practice (CoPs) are a collaborative knowledge transfer strategy that can be used for evidence-based practice implementation. The aim of this study was to describe the components and evaluate a CoP for speech-language pathologists about participation and aphasia rehabilitation.

**Methods:**

Logic analysis was used to determine the adequacy between resources, implemented activities, outputs and short-term outcomes of the CoP. Qualitative and quantitative descriptive data were collected through observation and participants’ logbooks. Outputs and outcomes of the CoP were revealed through thematic analysis and interpretation of descriptive statistics.

**Results:**

Resources including CoP design and educational aims, human and material resources were combined to create various web-based, online and offline activities. Participants invested more time per week than expected in the CoP, shared and created clinical tools and appreciated the array of suggested activities. Participant engagement allowed them to reflect, interact and collaborate with each other. All 13 participants reported they acquired knowledge about clinical tools and 12 mentioned they reflected on their practice. While the CoP was ongoing, six participants noticed evidence-practice gaps, seven prepared to change their practice, and three changed their practice towards including more participation-based considerations.

**Conclusions:**

This study showed that speech-language pathologists can include more participation-based approaches in aphasia rehabilitation as a result of participating in a time-bound, web-based CoP.

**Electronic supplementary material:**

The online version of this article (10.1186/s12913-019-4338-0) contains supplementary material, which is available to authorized users.

## Background

Aphasia is an acquired communication disorder following brain injury affecting not only expression and understanding of spoken language, reading, and writing [1], but also different aspects of participation, such as interpersonal interactions and relationships, major life areas, and community, social and civic life [2]. Although the ideal end purpose of rehabilitation is participation according to the United Nations [3], current aphasia rehabilitation practices may lack sufficient consideration of participation [4–6]. The International Classification of Functioning, Disabilities and Health defines participation as “involvement in a life situation” [2]. The participation of persons who have aphasia can remain limited after rehabilitation [7, 8], indicating a need that speech-language pathologists be more aware of evidence-based recommendations that promote participation and include these in their practice. The current paper reports on the structure, processes and outcomes of a knowledge transfer strategy for speech-language pathologists.

In Quebec’s continuum of publicly funded health care system (Canada) [9], stroke survivors are first admitted to an acute care hospital, where they generally stay between five days and three weeks, depending on the severity of the stroke, and receive healthcare and early short-term rehabilitation services [10]. Once their medical condition is stable, they either return home or attend an inpatient rehabilitation centre, where they receive intensive rehabilitation services generally five days a week for an average of 6–12 weeks, with longer rehabilitation associated with more severe strokes [10]. Where available, outpatient rehabilitation services are usually offered at a lesser weekly frequency and for an average of three months. However, there are no long-term rehabilitation services available in Quebec, reinforcing the importance of addressing participation early in the care continuum. Some persons with aphasia will join community associations which provide group leisure and social activities, but these are not available in all regions of Quebec [11].

Speech-language pathologists are encouraged to follow evidence-based practice (EBP) recommendations [12–14] across the care continuum of aphasia rehabilitation [15–17]. In aphasia rehabilitation, there is evidence that therapy improves reading, writing, expressive language, and “functional” communication, i.e. successful communication of a message via spoken, written, or non-verbal modalities, or a combination of these, in day-to-day interactions [1]. However, some speech-language therapy clinical activities may also have a positive impact on the level of participation of persons with aphasia after rehabilitation. Such clinical activities include, but are not limited to: setting goals based on the needs of the persons with aphasia and those of their relatives [18–20]; assessing and evaluating the outcomes of therapy with tools that measure participation (e.g. *Community Integration Questionnaire, Assessment for Living with Aphasia*) or quality of life (e.g. *Stroke Specific Quality of Life Scale*) [21–23]; training conversation partners to use communication strategies with the person who has aphasia [24, 25]; teaching persons with aphasia to use alternative methods of communication, such as gestures, writing, and/or pictures [26]; and openly discussing discharge plans from speech-language pathology services with the person with aphasia [27–29]. In the context of the present work, such clinical activities were referred to as “participation-based aphasia rehabilitation”, an expression that reflects rehabilitation practices focused especially on a person’s needs as they relate to participation. Participation-based aphasia rehabilitation may share characteristics with what was described in the literature as “comprehensive” aphasia therapy, such as Intensive Comprehensive Aphasia Programs (ICAPs) and the Life Participation Approach to Aphasia (LPAA) (e.g. [30–32]). Participation-based aphasia rehabilitation is not widely used in current speech-language pathology practice [4–6]. Therefore, effective knowledge transfer strategies are needed to support speech-language pathologists in employing intervention strategies that impact participation, the ideal end purpose of rehabilitation.

Rehabilitation speech-language pathologists experience challenges when implementing EBP [14, 33] especially since they report limited time for participating in EBP activities [14, 15, 34–36]. These challenges may also be related to the *nature*, or strength, of the evidence, i.e. the fact that despite randomized controlled trials and systematic reviews being highly valued in research, few are available in speech-language pathology [14]. Furthermore, applying EBP is problematic because of the *use* of evidence, i.e. speech-language pathologists have difficulty transferring the results of scientific research in their clinical reality, and may prefer consulting their peers for clinical decision making [14, 36]. Thus, an effective knowledge transfer strategy should be time-efficient, present clinically pertinent evidence and provide opportunities for collaboration with peers.

Collaborative knowledge transfer strategies offer a high level of interaction among participants, and between participants and researchers [37]. Communities of practice (CoPs) can be used to transfer knowledge. A CoP is defined as a “group of people who share a concern, a set of problems, or a passion about a topic, and who deepen their knowledge and expertise in this area by interacting on an ongoing basis” (p.4) [38]. To arouse interest among potential participants, a CoP should have educational aims and contribute to continuing education. Moreover, when conducting research about a CoP, one needs to operationalize some design aims to orient data collection [37]. In fact, in aphasia rehabilitation, CoPs for speech-language pathologists were never studied.

A systematic scoping review indicated that among the many reasons for undertaking the evaluation of a CoP there are the need to assess, to understand, and/or to promote their value [39]. Assessment frameworks of CoPs frequently include attention to the goals, context, structure, process/activities, outcomes, and level of impact [39]. CoPs can thus be analyzed with a logic model, traditionally including *resources/inputs*, *activities*, *outputs*, and *outcomes/impacts* [40–45]. *Resources* include human and material resources and other inputs to support the intervention; *activities* refer to all necessary action steps to produce the outputs; *outputs* are the products of the intervention; and *outcomes* are changes resulting from activities and outputs [44]. Logic analysis allows one to create a model illustrating the different components of an intervention and the relationships between them [46], thus it was an appropriate method for evaluating the current CoP. This method was chosen because of its potential to detail each component of the CoP [40] and to describe outputs and outcomes while it was ongoing and especially afterwards to evaluate the CoP’s immediate success.

Four theoretical concepts were used for the design and implementation of this CoP: constructivism, social learning, situated learning, and reflective practice. Constructivism postulates that meaning and experience come from social interaction and that knowledge is co-constructed by the learners [47]. Social learning theory posits that knowledge is best integrated through social interaction [48]. Situated learning refers to learning in authentic and meaningful contexts [49]. Reflective practice is the product of a tacit and implicit knowledge in one’s actions [50]. In speech-language pathology, reflective practice can be operationalized by conducting two types of activities: written reflection and reflective discussion [51].

Given that aphasia affects a person’s level of participation and that participation is the under-achieved ideal end purpose of rehabilitation, a CoP about participation-based aphasia rehabilitation in line with current best evidence was designed, implemented and evaluated using logic analysis. Therefore, the aim of this study was to describe the components and evaluate a CoP for speech-language pathologists in aphasia rehabilitation.

## Methods

This section presents details about the context of the study, topics of the CoP, participants, data collection and data analysis.

### Context

This study was undertaken as part of the first author’s PhD research (CAG). GLD was CAG’s advisor. Speech-language pathologist participants were recruited to be part of a CoP. They were aware that the CoP was evaluated and knew they could use their involvement as professional continuing education. The CoP was presented to the participants as an opportunity to learn about EBP recommendations, to reflect on their practice and to interact with colleagues. Its ultimate and explicit aim was to fill a gap between literature about participation-based aphasia rehabilitation and actual practices. It is possible that such a gap existed because clinical practice guidelines were not of sufficient quality in the field of post-stroke aphasia [26, 52], engaging in the uptake of these guidelines was too time intensive [53], or they were not pertinent to speech-language pathologists’ specific work settings or clients. Finally, the CoP was web-based to allow the participation of speech-language pathologists from all regions of Quebec.

### Topics of the CoP

In Quebec, speech-language pathology practice usually involves assessing language, voice, and speech functions, in order to elaborate a treatment plan, and conduct indirect and direct intervention to facilitate a person’s communication in his/her environment [54]. No specific details or evidence were available in relation to specific clinical activities that should be provided in the Quebec healthcare context. In contrast, the *Australian Aphasia Rehabilitation Pathway* (AARP) [55] presents speech-language pathology practice as a pathway composed of the following evidence-based clinical activities: receiving the right referrals, optimizing initial contact, setting goals and measuring outcomes, assessing, providing intervention, enhancing the communicative environment, enhancing personal factors, and planning for transitions. Topics were selected considering available evidence, the potential they had to induce reflective practice, and because they were part of the usual clinical activities of speech-language pathologists. Furthermore, CAG conducted individual interviews with all participants before the beginning of the CoP. These interviews allowed researchers to understand and describe the practice of Quebec’s speech-language pathologists in terms of central and peripheral clinical activities [56] and informed the design of the CoP. For the purposes of a time-bound PhD research project, the CoP was “top-down” and created by the researchers, who selected the topics. However, these were chosen to mirror participant clinical realities and interests. These topics were: 1- the ideal end purpose of aphasia rehabilitation; 2- goal-setting; 3- assessment and outcome evaluation; 4- indirect intervention; 5- direct intervention; and 6- discharge.

### Participants

Fourteen speech-language pathologists with a minimum of six months of experience in aphasia rehabilitation were initially recruited to participate in the CoP. One left the project for professional reasons after two weeks, and the data from this participant was not analyzed. The 13 remaining participants (12 women, one man) from the Greater Montreal area (*n* = 9) and suburban regions (*n* = 4) of the province of Quebec worked in various aphasia rehabilitation settings: acute care, inpatient, and/or outpatient rehabilitation (see Table 1). Three participants had work experience in a privately funded – but free of charge for users – community-based service for family members of persons with aphasia, and two had worked part-time in a community association for persons with aphasia.

**Table 1:** Demographic information about the 13 CoP participants

No of participants	
*Age range (min = 25; max = 53; mean = 37.9 yrs)*	
25–34	6
35–44	3
45–54	4
*Years of experience in speech-language pathology (min = 1.5; max = 28; mean = 12.2)*	
< 5 years	6
> 14 years	7
*Main work setting*	
Acute care	2
Inpatient rehabilitation	5
Outpatient rehabilitation	6
*Terms of participation in the CoP*	
Dedicated paid time for the whole project	3
Dedicated paid time for part of the project	8
No dedicated paid time	2

The researchers recruited participants from various settings to engage individuals across the aphasia care continuum who could share their unique experience [57] and to ensure rich and varied perspectives and discussions. CAG presented the project to the speech-language pathologists of three rehabilitation centres affiliated with the team’s multi-site research organization and recruited six participants. Three were informed about the project by their clinical supervisor. Two were recruited via emails sent to other institutions of the aphasia rehabilitation care continuum. Finally, two participants contacted CAG directly after they heard about the project. Participants demonstrated their interest by signing the consent form which detailed the topics of the CoP and the expected time investment. Three participants were colleagues of CAG, and seven others had previously interacted with CAG, GLD or their collaborators in various research projects. The other participants had no specific relationships with the university or researchers.

### Data collection

Data was collected through the web platform which recorded the participants’ activities and through weekly logbooks that participants completed. Participants were aware of the engagement of others in most suggested activities of the web platform, which was a prerequisite for interaction among participants. Logbooks were designed to help CoP members reflect on their contributions and learning. They contained questions pertaining to time spent in each activity and/or for the whole week, appreciation of activities, and perceived outcomes. Filling logbooks was an individual activity, and the content of logbooks was not shared with other participants. A logbook document is available in Additional file 1.

### Data analysis

Data analysis was guided by logic analysis, which helped describe and illustrate the relationships between all components of the CoP. Thematic analysis and descriptive statistics were undertaken simultaneously and informed the creation of the logic model, which was updated and refined as analyses progressed.

#### Logic analysis

In order to determine the relationships between the problem, the CoP, and its potential ultimate benefit, a logic model was created [40] that represented the resources, activities, outputs, and outcomes. General concepts about CoPs previously mentioned guided the establishment of the design and educational aims of the CoP. The combined expertise of CAG and GLD was another resource that informed the CoP’s content and activities. The participants’ engagement in the activities provided the outputs and outcomes, including quantitative and qualitative data. After data collection, logic analysis was chosen to provide an analysis framework because of its potential to faithfully represent the results [40].

#### Thematic analysis

Thematic analysis was performed on the written content of the logbooks using NVivo for Mac 11.4.1 to provide a rich and detailed account [58] of the participants’ appreciation, participation, and perceived outcomes of the weekly activities. Inductive data-driven thematic analysis involved: 1- familiarizing oneself with the data, i.e. reading the logbooks’ extracts several times and in different orders over a period of six weeks; 2- generating initial codes, i.e. coding features of the data inductively and across the entire data set; 3- searching for themes, i.e. grouping together codes conveying similar meaning into potential categories, which were later grouped together into themes and subthemes; 4- reviewing themes and subthemes; 5- refining the analysis as part of an iterative process through regular and repeated discussions between CAG and GLD; 6- confirming the relationship between extracts, themes, and subthemes; 7- defining and labelling themes and subthemes; and finally 8- translating them from French (language in which the data was available) to English, with careful attention to retain the precise meaning of each theme and subtheme [58]. Extracts from the logbooks were also translated to English.

#### Descriptive statistics

Descriptive statistics were used to summarize the quantitative data collected in the logbooks and recorded on the web platform, i.e. the number of collected logbooks, the number of participants per activity, the number of minutes spent each week in the CoP, and the number of minutes spent per activity.

## Results

**Fig. 1:**
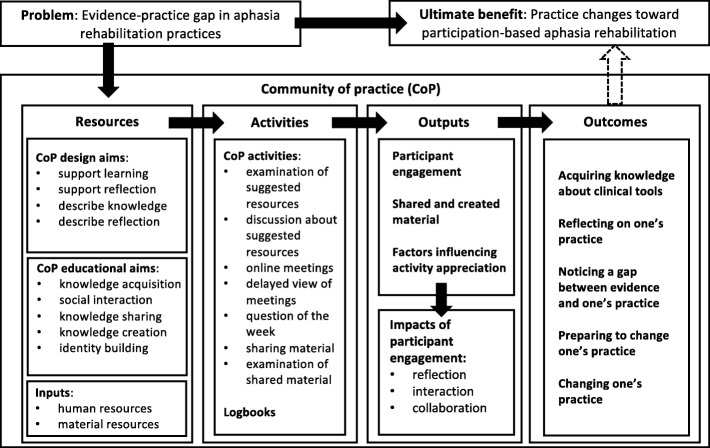
Representation of the community of practice using the logic model

This section describes the logic model of the CoP and details its resources, activities, outputs, and outcomes (see Fig. 1). Each component of the CoP was critically considered through the lens of logic analysis. Outcomes were described using thematic analysis of 316 logbook entries, which varied in length between one word and a few sentences.

### Resources

Resources included design aims, educational aims, and inputs of the CoP, detailed below.

#### CoP design aims

The CoP was designed to 1- support learning among participants; 2- support reflection among participants [38]; and ultimately it aimed to collect data to 3- describe outcomes in terms of knowledge acquired; and 4- describe participant reflections related to the topics of the CoP.

#### CoP educational aims

Five educational aims were considered: 1- knowledge acquisition, as per Wenger’s definition of a CoP [38]; 2- social interaction; 3- knowledge sharing; 4- knowledge creation; and 5- identity building, as per the main characteristics of a CoP in health care [59]. Table 2 shows the relationship between educational aims and the CoP activities. Most educational aims were related to specific activities, but knowledge creation and identity building had the potential to be achieved through any or all of the activities. When designing the CoP each activity was explicitly related to one or several educational aims.

**Table 2:** List of the CoP educational aims and related activities

CoP educational aims	Activities
Knowledge acquisition	Examination of suggested resources
Social interaction	Discussion about suggested resources
	Online meetings
	Delayed view of online meetings
	Question of the week
Knowledge sharing	Online meetings
	Sharing material with others
	Examination of shared material
Knowledge creation	Potentially all activities
Identity building	Potentially all activities

#### Inputs

Human and material resources were deployed for this CoP. Participants were considered a resource, because they had varied experience and dedicated paid time to be involved in the CoP (see Table 1). CAG was the CoP facilitator and invested many hours per week to prepare the upcoming activities. She led the online meetings, managed the web platform, sent weekly emails to participants, and answered participants’ questions. GLD, a Professor in speech-language pathology, participated in the design of the activities and provided expertise about resources in participation-based aphasia rehabilitation and teaching skills. She was present at the online meetings to take notes as a basis for discussion with CAG. A research assistant took care of technical aspects of the online meetings (e.g. turning on/off participants’ microphones, providing written answers in the forum for those who wrote questions during meetings, etc.) The chosen user-friendly platform for the CoP was *Adobe Connect*.

### Activities

A first meeting on the web platform allowed participants to get to know one another and be exposed to the theoretical concepts underlying the CoP. Twelve participants were online during this meeting, including one who had technical problems and watched the part she missed afterwards. The other participant was not available at the time of the meeting and viewed it later.

During the initial meeting, participants were asked to invest about 1 h per week over the six weeks of the CoP in one or several of the suggested activities. They could spend this time on any activity they chose and considered interesting. The time commitment needed was estimated on the basis of what seemed a reasonable amount of time spent in addition to regular clinical activities. Most participants were paid by their employer for 1 h a week for their time investment in the CoP. This hour was included in their regular work schedule and they did not receive extra payment for participating. They were not expected to participate in all of the activities every week and there were no requirements regarding the number of activities participants should engage in weekly. One week of break was provided after the three first weeks during which participants could catch up if needed. Also, during the initial meeting, participants expressed the desire to have more online meetings than initially planned, and a further schedule of four meetings was arranged.

An array of seven types of activities were initially planned and offered to CoP members: online meetings about the weekly topic; viewing the recorded meetings; examination of suggested resources (i.e., clinical tools for goal-setting, assessment, and intervention including research articles, websites, and standardized tests); an interactive discussion forum about these resources; an interactive discussion forum related to a weekly question; a folder to share material used in one’s clinical practice; and examination of materials shared by others. One-hour online topic discussions were offered four times for the following topics: 1- goal-setting; 2- assessment and outcome evaluation; 3- indirect intervention; and 4- discharge. During online meetings, CAG asked general questions about the topic, gave the floor to those who wanted to contribute, occasionally summarized what had been said, and facilitated the discussion without giving prescriptive information. She did not share her personal opinion on the topic, which allowed participants to freely interact, co-construct the meaning of what they were learning and co-create knowledge. Offline activities were available each week. Researchers offered a total of 32 resources for participants to examine and discuss, at their leisure. The “question of the week” concerned the weekly topic (e.g. “How can discharge from rehabilitation be a positive experience for your client, your client’s family, and yourself?”). Each week, participants were asked to complete a logbook summarizing their involvement in the CoP. Logbooks were reflective tools that provided information about CoP participation, outputs and outcomes.

### Outputs

The outputs described in this section are: the level of participant engagement, the material shared and created by participants, the factors influencing the participants’ appreciation of the activities of the CoP, and the impacts of participant engagement.

#### Level of participant engagement in activities

The 13 participants completed 71 logbooks over six weeks with a mean of 11.8 logbooks per week. The time spent in the CoP remained approximately constant overall, with a mean of 101 min invested per week per participant, and a mean of 16 h overall (min = 12; max = 24), including the initial and final online meetings, a relatively higher investment of time than the 60 min anticipated in the design of the CoP. Eight participants spent more time in the CoP than the clinical time allowed to the project by managers and two participated without having dedicated clinical time, indicating the participants’ high level of interest. For each weekly topic, there were between three and 11 participants who engaged in three or more suggested activities, indicating a very high level of engagement. On weeks 1, 2 and 6, all participants engaged in at least one activity. Week 2, about goal-setting, was the most popular in terms of time spent and number of completed activities. Moreover, the online topic discussion held in week 2 had the highest number of participants. Week 5, about direct intervention, appeared to be the least popular, both in terms of time spent and number of completed activities (see Table 3).

**Table 3:** Quantitative data related to participation in the activities as a function of the weekly topic of the CoP

Week	Topic	No of collected logbooks (/13)	No of participants investing in 3 or more activities	No of participants investing in 1 or 2 activities	No of inactive participants	No of participants viewing or involved in online meetings	Mean duration of investment (minutes)
1	Ideal end purpose of aphasia rehabilitation	13	7	6	0	–	106
2	Goal-setting	12	11	2	0	11	106
3	Assessment and outcome evaluation	13	8	4	1	8	100
4	Indirect intervention	11	5	5	3	6	106
5	Direct intervention	10	3	7	3	–	90
6	Discharge	12	4	9	0	9	95

Participants invested the most time in the online discussions (or viewing the recordings if participants were unavailable when they were held), which was about 1 h for each meeting. They spent an average of almost 1 h each week examining the suggested resources and preparing clinical material to share with CoP members. All participants took part in or viewed at least one online meeting, examined one or more suggested resource, answered at least one weekly question, and examined shared material. The participants most frequently engaged in: answering the question of the week, examining suggested resources, and participating in online meetings. Even when engaging in offline activities, such as answering the question of the week, participants interacted with one another, through writing rather than in conversation. Interaction in the written modality was slower and probably involved reflection when reading and responding to someone else’s comments. Considering the time invested and the number of participants engaged in them, the most popular activities were judged to be the online meetings, their delayed view, and the examination of suggested resources. The least popular activity was the discussion about the suggested resources. In general, logbooks tended to underestimate the level of engagement in the CoP, in that participants sometimes forgot to note in the logbook an activity they did, while the platform had recorded that they had engaged in that activity. For example, participants had indicated in their logbooks that they attended the online meetings a total of 21 times, whereas they actually had attended 24 times (see Table 4).

**Table 4:** Quantitative data related to number of weeks the activity was offered, number of logbooks, participant engagement in activities, and duration of engagement in activities

Activity type	No of weeks the activity was suggested	Total No of logbooks reporting on the activity	Total No of times participants engaged in the activity	Total No of participants engaged in the activity	No of participants engaged in the activity 3 times or more	No of participants engaged in the activity 1 or 2 times	No of inactive participants	Mean duration in minutes per week^c^ (min-max)
*More popular activities*								
Initial online meeting^a^	1	12	12	12	–	–	–	–
Thematic online meetings^a^	4	21	24	11	4	7	2	61 (60–75)
Delayed view of meetings^b^	4	9	9	8	0	8	5	60 (60–60)
Examination of suggested resources^b^	6	32	32	13	9	4	0	56 (5–150)
*Less popular activities*								
Discussion about suggested resources^a^	6	6	17	7	3	4	6	15 (5–30)
Question of the week^a^	6	25	42	13	7	6	0	19 (5–60)
Sharing material with others^a^	6	7	21	10	3	7	3	53 (20–90)
Examination of shared material^b^	6	24	24	13	8	5	0	20 (10–60)

^a^Data regarding participation in these activities came from researchers’ direct observation on the platform

^b^Data regarding participation in these activities came from entries in participants’ logbooks

^c^Data regarding time invested in activities came from entries in participants’ logbooks

#### Shared and created material

Thirty-eight documents were shared in total on the dedicated module of the platform. They included clinical success-stories written by participants, clinical tools (e.g. standardized tests, informal assessment material, therapy material), research articles they had read, summaries of research articles or conferences, material produced by participants for training purposes, and a poster presentation one participant had prepared. Also, during two separate online meetings, participants brainstormed ideas about clinical tools that did not exist and that would be useful for practice. After these meetings, two different participants wrote up the ideas discussed and spontaneously elaborated two co-created tools: a framework for client goal-setting and a checklist of strategies for communication partners.

#### Factors influencing activity appreciation

In their logbook comments, participants generally reported to what extent they appreciated the activities. The thematic analysis of 186 comments extracted from the logbooks’ sections “Describe your appreciation of the activity” and “Describe your participation” (see Additional file 1) revealed four factors influencing the degree of appreciation of the different activities. These factors were: the time they had to do the activities, the level of interaction between participants, the format, and the content of activities (see Table 5). Overall, positive comments outnumbered negative comments and the number of satisfied participants exceeded the number of dissatisfied ones for all factors except time. Participants would have dedicated more time on CoP activities if they had had more clinical time to devote to the CoP. Also, they were mostly satisfied with the high level of interaction and collaboration offered by some activities, appreciated the different ways to participate, and characterized the activities as “interesting”, “instructive”, “pleasant”, and/or “pertinent” for their clinical practice.

**Table 5:** Themes related to the factors influencing appreciation of the CoP activities, extracts from logbooks, number of extracts per theme and number of participants mentioning each theme

Factors influencing appreciation of activities	Extracts (*n* = 186)	Total No of extracts per theme	Total No of participants mentioning the theme
*Time available to do the activity and reflect*			
- Appreciating to take the extra time	*I appreciated this moment because I took the time to sit down, read and take notes. This allowed me to take my reflections further than usual.*	9	5
- Experiencing a lack of time despite a high interest	*Once again, I printed journal articles that I still have to read because they looked interesting. Time was short!*	22	8
*Level of interaction and collaboration between participants*			
- Being satisfied with a high level of interaction	*I had to think before speaking* [during online meetings]*, express my opinion, which pushed my reflection further. I found it so rewarding to talk with others who also reflected a lot.*	30	8
- Finding that some activities offered limited interaction among participants	*The interaction among participants was limited because we did not necessarily read the same journal articles* [discussion on suggested resources].	13	3
*Format of the activity*			
- Appreciating the efficiency of the activities and the different ways to participate	*I had the opportunity to share my opinions at the time I wanted to share them* [during the initial online meeting]*. I appreciated the fact that I could participate by using the microphone or by writing in the forum.*	29	10
- Experiencing technical or format-based difficulties with the platform and activities	*I could not fully participate in the discussion* [because of a connexion problem].	14	9
*Content of the activity*			
- Considering that some activities were interesting and pertinent	*The material was interesting because it was directly applicable to my clinical reality.*	63	13
- Considering that some activities were less useful	*I briefly looked at* [the suggested resources]*; they were too general for me.*	6	4

#### Impacts of participant engagement

As a consequence of engaging in the various CoP activities, sharing and co-creating material, and appreciating the activities they engaged in, participants had many opportunities for reflection, interaction and collaboration. They reflected about their practice among a group of peers, interacted on a regular basis with colleagues, and collaborated in the elaboration of material and ideas. All of the observed outputs and the impacts of participant engagement led to the outcomes of the CoP, described in the next section.

### Outcomes

The content analysis of 130 comments extracted from the logbooks’ sections “Describe your learning outcomes” and “Other comments” (see Additional file 1) revealed five themes, further described below: 1- acquiring knowledge about clinical tools; 2- reflecting on one’s practice; 3- noticing a gap between evidence and one’s practice; 4- preparing to change one’s practice; and 5- changing one’s practice (see Table 6).

**Table 6:** Themes describing outcomes of the CoP, extracts from logbooks, number of extracts per theme and number of participants mentioning each theme

Outcomes	Extracts (*n* = 130)	Total No of extracts per theme	Total No of participants mentioning the theme
*Acquiring knowledge about clinical tools*	*I learned about the* Better Conversations with Aphasia *web site, which I did not know about.*	34	13
*Reflecting on one’s practice*			
- Reflecting on topics of the CoP	*I think that group therapy is an interesting avenue. It is close to normal life and allows one to meet other people. It can reduce isolation and directly enhance social participation.*	21	10
- Building identity through interaction	*I realized that we all aim for social participation somehow.*	37	11
*Noticing a gap between evidence and one’s practice*	*I lack the structure that would reveal the expectations and goals of the patients and those of their families.*	8	6
*Preparing to change one’s practice*	*After reading* Sherratt et al., 2011*, I realized that giving information about aphasia and its related difficulties to the person with aphasia and his/her family could become a more important therapy goal in my acute care practice.*	18	7
*Changing one’s practice*	*This* [an online meeting] *inspired me to create a general document for goal-setting with the patient and to give it to him/her afterwards. I used it for the first time today with one patient: it was super interesting. As she lives by herself, I never really talked about her relatives but after reading the article* [Hersh et al., 2012] *and creating a checkbox labelled “Relatives” in my goal-setting document, it brought many interesting points, including the relatives’ need to have more information about aphasia.*	3	3

All participants acquired knowledge about clinical tools as a result of this CoP. These clinical tools included research articles, websites, tests, material for intervention, practice guidelines, and resources available in the care continuum of aphasia rehabilitation. Twelve participants described their reflections about their practice. Reflections concerned the topics of the CoP and how the new knowledge had effects on their perception of their practice (e.g. acknowledging that group therapy has many advantages) and were indicative of identity building through interaction with other CoP members, for example in realizing that other members experienced similar challenges in their practice. Participants explored their own thoughts about one aspect of clinical practice, compared their clinical activities with those of others, validated their approaches through discussions, and realized there was a certain consistency across settings*.* As recorded in logbooks, six participants noticed a gap between the evidence on participation-based aphasia rehabilitation available through the CoP and one or more aspects of their clinical practice. Seven participants mentioned they were preparing to change an aspect of their clinical practice in line with what had been discussed in the CoP. These probable changes included: using a new participation-based clinical tool, such as a structured questionnaire to establish personalized goals with the client; integrating in therapy a recommendation reported on a website or journal article, such as the SMARTER goal-setting framework [19]; being more systematic in presenting information to clients about community services; measuring therapy outcomes related to the person’s life habits; training conversational partners; and including relatives more frequently in interprofessional team meetings. Finally, during this CoP, three participants reported they were now using a new participation-based clinical tool. For example, the participant who gathered the ideas about the co-created goal-setting framework reported in her logbook that she was now using it. It was not expected that participants would make changes to their clinical practice while the CoP was ongoing. There was no clear relationship between the engagement of participants in certain topics or activities and the types of outcomes they reported.

In summary, human and material resources were made available for this web-based CoP, that was designed with a number of desirable educational aims in mind. Logbook data were collected and analyzed as well as the information about participants’ engagement in the online and offline activities recorded on the platform. Participants invested more time than the suggested 1 h per week. They shared many clinical tools and co-created two new ones. All 13 participants expressed significant outcomes of the CoP on their practice, such as learning about clinical tools, reflecting on their practice, and/or considering or trying out new ways of providing participation-based aphasia therapy (see Fig. 1). There was no indication that participants from across the aphasia care continuum considered that participation-based rehabilitation was not appropriate for their specific clients. There was no evidence that the type of workplace was related to a type of outcome reported by participants.

## Discussion

The aim of this study was to describe the components and evaluate a CoP for speech-language pathologists in aphasia rehabilitation. This study provides evidence that a six-week structured CoP can facilitate learning, reflection, interaction with peers, and induce short-term changes in speech-language pathologists’ aphasia rehabilitation practice. The results are consistent with the *Readiness to change* framework [60], which suggests clinicians can contemplate change, prepare to change, or actualize changes in the practice, thus indicating that most participants were somewhere along that axis. Logic analysis helped to describe the components, their relationships and the related short-term effects of this CoP [40–46], thus contributing to the literature about CoPs for promoting EBP in the health care sector [59, 61]. The reasons for the apparent success of this CoP are numerous.

This CoP was successful because of the continuous opportunities for reflection, interaction, and collaboration. All participants achieved the three components of participant engagement. Thus, logic analysis revealed the importance of participant engagement in the CoP activities and in achieving its aims. According to social learning theory, knowledge acquisition better operates in groups such as CoPs [48]. Reflective practice is also easier in groups of peers [62], and in small groups of peers when it comes to speech-language pathologists [51]. Reflecting in a group allowed participants to interact. Participant interactions led to collaboration and creation of clinical tools, which have a high potential to be used in clinical settings [63]. Creating knowledge is consistent with the concept of constructivism [47], in which it is recognized that knowledge is generated through the experience of participants. This was achieved within this CoP, as shown by the new material that emerged from participant collaboration. Also, the CoP provided opportunities for participants to write down what they learned and share their thoughts with others, thus facilitating reflective practice [51]. Various factors may explain the impacts of participant engagement and ultimately contributed to the CoP’s apparent success.

Participants appreciated the variety of ways they could partake in the CoP, in line with their preference and/or availability for interactive activities or for individual reflection. Results do not allow us to report on the factors that influenced the participants’ specific interest for non-interactive activities. However, participants learned about new clinical tools when consulting suggested resources on the platform. There is evidence that offline individual CoP activities can be as equally effective [64] as interactive activities. Participants stated both types of activities were useful for their clinical practice, and this possibly helped to arouse and maintain interest and motivation throughout the CoP.

The positive outcomes of this CoP could be partially explained by the fact that the participants were particularly motivated and interested by the suggested topics. Some participants, who already knew the researchers’ interest in participation and aphasia rehabilitation, were maybe more informed than other CoP members about the topic. Also, participants who had worked in community services for persons with aphasia or their relatives were possibly more sensitive than others to certain topics, such as conversation partner training. These participants were potentially more prone than others to question their own practices and more motivated to engage in the CoP. Motivated speech-language pathologists have a higher potential to change their practice [65], which may explain some of the results. Less motivated individuals may not experience the same benefits. Unfortunately, it is not possible to determine retrospectively all of the motivational factors at play in this CoP, especially factors which were not conscious or explicit. Thus, the results may not be applicable to speech-language pathologists working in other settings, with different clienteles, or where rehabilitation services are not offered through a public system. The motivation of speech-language pathologists was more apparent for some topics than for others. The popularity of the goal-setting topic might be explained in part by the fact that this is a central clinical activity that helps determine the following ones [19], added to the fact that some participants were exposed for the first time to the possibility of considering goal-setting before assessment [55]. Goal-setting was also the topic of the first online topic discussion. The low level of engagement in the topic of direct intervention might be explained by the fact that it was presented in the second to last week of the CoP, that there was no online meeting on that week, and/or that participants did not feel the need or were not ready to reflect on the activity that dominates their daily practice. In fact, the popularity of the topics was probably influenced by many interacting factors.

In this study, most participants enjoyed some financial support for participating in the CoP which certainly contributed to their level of motivation and the resulting time spent in the CoP. In the literature, time is often cited as a barrier to implement EBP or practice changes [14, 15, 34–36]. The time participants took to reflect, interact and collaborate with others, although insufficient to the liking of the participants, was quite high and allows us to think that the time barrier was surmounted at least partially, since the participants provided evidence of emerging and further practice changes. Indeed, time does appear to be a significant factor in the adoption of new practices related to the best evidence. The discrepancy between the high amount of time devoted to the CoP and participants’ perceived lack of time resonates with a conflict clinicians experience between time spent in knowledge transfer activities and time spent delivering direct services to clients [66]. This conflict was not addressed and persisted throughout the CoP. One way this internal conflict could have been attenuated would have been to enlist more organizational support [34] and as much dedicated paid time as participants actually devoted. But such a state of affairs was unlikely to happen in a financially restricted health care system. Furthermore, when CoPs are time-bound, like in the present case, attrition is reduced [67] and participants are mobilized for a specific period of time which may not occur in ongoing CoPs. Finally, overall interest, motivation and involvement may have helped the participants who did not have dedicated clinical time to also engage highly in the CoP.

It is also possible that this CoP provided participants with a new and different opportunity for continuing professional development. Participants who did not live in the greater Montreal area were appreciative of the fact that they were not expected to travel to participate. Moreover, young and experienced participants reported that they were excited to participate in a research project about their clinical practice that could provide them with immediate professional benefits.

The basic characteristics of successful CoPs – social interaction, knowledge sharing, knowledge creation, and identity building [59] – were considered: participants had repeated opportunities to interact, share and create clinical tools, and may have constructed their professional identity. Although it is not clear how one should define and measure the construction of professional identity, participants indicated that they had compared their practice to that of other members, and that the experience of other members was a form of validation of their own practice. This indicates that speech-language pathologists benefitted from interaction to define their practice [36]. The concept of “identity building” could be further explored.

### Limitations

This *top-down* CoP was created by researchers whereas most CoPs are frequently *bottom-up*, i.e. created by the members themselves*. Bottom-up* CoPs are considered more efficient because the members’ needs drive their creation [68], but the success of the present CoP suggests that it answered some of the speech-language pathologists’ needs, even though they were not explicitly expressed. Critically, the first author was a practicing speech-language pathologist who was aware of the limitations of her own practice in achieving optimal participation for persons with aphasia. She had a professional interest in sharing and exploring participation-based approaches, which participants may have recognized implicitly as similar to their own desire to improve their practice. Also, a great investment was required on the part of CAG and GLD, who had previous specific knowledge in the area of participation-based aphasia rehabilitation, to design, implement and facilitate the CoP. Since this project was part of the first author’s PhD research, she was motivated in supporting the participants in achieving the goals of the CoP, as was her advisor, GLD. However, the CoP could not be maintained after the end of the project since it was planned to be a time-bound CoP. Nevertheless, the CoP template, as originally designed, with the materials and activities planned out could be used again requiring a much smaller commitment to facilitate the activities.

### Future studies

Although positive outcomes were demonstrated, future studies are needed to validate these results and evaluate the long-term outcomes of the CoP. Future studies should also help to determine which professional environments are most conducive to learning and implementation of some of the participation-based intervention tools presented and discussed. Participants did not all have the same level of comfort or ease in the online activities. They appreciated the choice of investing time in online or offline activities. Thus, we believe that participants of future CoPs should continue to benefit from a choice between online and offline activities. Moreover, participants may have reported more positive outcomes than challenges in their logbooks, which may not reflect all of the participants’ issues, learning, and global experience in the CoP.

The role of this intensive but relatively short time-bound CoP in professional identity development of speech-language pathologists should be explored with respect to long-term outcomes on practice. To have impacts on practice, it is possible that some key ingredients such as engaged peers interacting and collaborating on issues of clinical interest are critical in a CoP, more so than a specific duration of participation. Further studies are needed to yield information about the role, structure and processes of CoPs in stimulating practice changes and continuing professional identity development.

## Conclusions

This study indicates that speech-language pathologists can include more participation-based approaches in their aphasia rehabilitation practice within a CoP intentionally designed to support learning, reflection, interaction and collaboration among peers.

## Additional file

Additional file 1: Example of a logbook for week 2. (DOCX 32 kb)

## Data Availability

The datasets generated in French and analyzed during the current study are not publicly available because individual privacy could be compromised. However, they could be made available given a reasonable request to the corresponding author and with further approval of the Ethics Committee, which would be specifically sought. Moreover, the documentation (in French) related to the design of the CoP, the resources employed, and the weekly activities could be made available given a reasonable request to the corresponding author.

## Abbreviations

CoP: Community of practice
EBP: Evidence-based practice
