# Supplementary material for: Evaluation of a community of practice for speech-language pathologists in aphasia rehabilitation: a logic analysis
Source: BMC Health Serv Res. 2019 Jul 29;19:530. doi: 10.1186/s12913-019-4338-0 (PMC6664764; doi:10.1186/s12913-019-4338-0)
Supplement: Supplementary file 1 — Example of a logbook for week 2. (DOCX 32 kb) [file 12913_2019_4338_MOESM1_ESM.docx]

Additional file 1 Example of a logbook for week 2^[[1]](#footnote-1)^

Week 2 Topic: Goal-setting

1. I visited the platform this week: yes  no

If not, could you share the reason?

1. Please answer the following questions regarding the weekly activities:

|  | I participated in the activity | Describe your appreciation of the activity | Describe your participation | Describe your learning outcomes | Other comments |
| --- | --- | --- | --- | --- | --- |
| Examination of suggested resources | yes no  Time: |  |  |  |  |
| Discussion about suggested resources | yes no  Time: |  |  |  |  |
| Question of the week | yes no  Time: |  |  |  |  |
| Examination of shared material | yes no  Time: |  |  |  |  |
| Thematic online meeting of [*Date*] | yes no  Time: |  |  |  |  |

1. Continued on next page [↑](#footnote-ref-1)
